# Supplementary material for: Large-Scale Mercury Dispersion at Sea: Modelling a Multi-Hazard Case Study from Augusta Bay (Central Mediterranean Sea)
Source: Int J Environ Res Public Health. 2022 Mar 26;19(7):3956. doi: 10.3390/ijerph19073956 (PMC8997764; doi:10.3390/ijerph19073956)
Supplement: Supplementary file 1 [file ijerph-19-03956-s001.zip › ijerph-1627337-supplementary.pdf]

International Journal of
Environmental Research and Public Health

MDPI

Supplementary materials

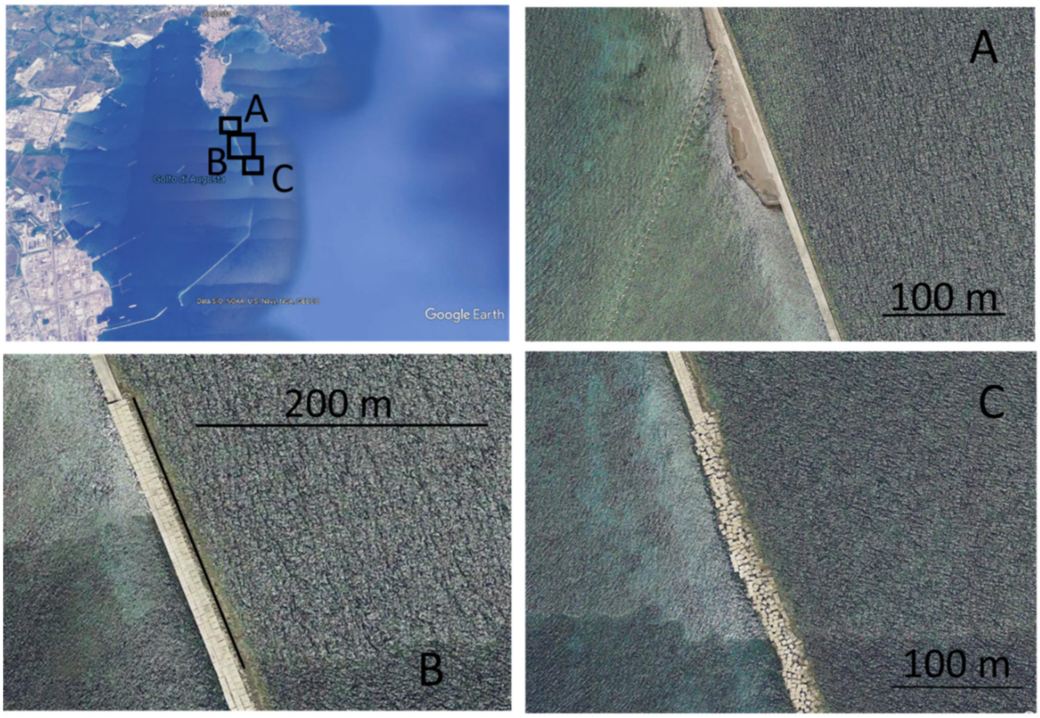

Figure S1. Breakwater system of the Augusta Harbor. The northernmost segment resulted to be composed by three different structures, i.e.: (from North to South, respectively) A), A concrete seawall (abouth 400 m length); B) a sequence of neatly arranged blocks (ca. 400 m length), and C), chaotically arranged concrete blocks (more than 1100 m; Figure S1 in supplementary materials). The other two segments that compose the breakwater have the same structure of C.
